# Management of burn injuries – recent developments in resuscitation, infection control and outcomes research

**DOI:** 10.1186/1757-7241-17-14

**Published:** 2009-03-11

**Authors:** David J Dries

**Affiliations:** 1 Regions Hospital, Department of Surgery, 640 Jackson Street, St. Paul, MN 55101, USA; 2 University of Minnesota, Department of Surgery, 420 Delaware Street S, Minneapolis, MN 55455, USA

## Abstract

**Introduction:**

Burn injury and its subsequent multisystem effects are commonly encountered by acute care practitioners. Resuscitation is the major component of initial burn care and must be managed to restore and preserve remote organ function. Later complications of burn injury are dominated by infection. Burn centers are often called to manage soft tissue problems outside thermal injury including soft tissue infection and Toxic Epidermal Necrolysis.

**Methods:**

A selected review of recent reports published by the *American Burn Association *is provided.

**Results:**

The burn-injured patient is easily and frequently over resuscitated with complications including delayed wound healing and respiratory compromise. A feedback protocol is designed to limit the occurrence of excessive resuscitation has been proposed but no new "*gold standard*" for resuscitation has replaced the Parkland formula. Significant additional work has been included in recent guidelines identifying specific infectious complications and criteria for these diagnoses in the burn-injured patient. While new medical therapies have been proposed for patients sustaining inhalation injury, a new standard of medical therapy has not emerged. Renal failure as a contributor to adverse outcome in burns has been reinforced by recent data generated in Scandinavia. Of special problems addressed in burn centers, soft tissue infections and Toxic Epidermal Necrolysis have been reviewed but new treatment strategies have not been identified. The value of burn centers in management of burns and other soft tissue problems is supported in several recent reports.

**Conclusion:**

Recent reports emphasize the dangers of over resuscitation in the setting of burn injury. No new medical therapy for inhalation injury exists but new standards for description of burn-related infections have been presented. The value of the burn center in care of soft tissue problems including Toxic Epidermal Necrolysis and soft tissue infections is supported in recent papers.

## Introduction

The burn-injured patient is unique in resuscitation requirements, metabolic stress, pattern of complications and determinants of outcome.[1] This review highlights a selected group of papers focused on those aspects of care which are unique to burn centers and the burn-injured patient and contribute in important ways to outcome.

Contemporary discussion of burn resuscitation features the Parkland formula proposed by Baxter and coworkers in the 1960s.[2] Reviews of recent experience with burn resuscitation suggest that treatment objectives and fluids administered in the approach recommended by the Parkland group are frequently exceeded.[3] What is contemporary thinking about initial fluid administration in the setting of burn injury? The *American Burn Association (ABA) *has recently presented a statement which begins to answer this question.[4] The Parkland Burn Center recently published a report on the use of the Parkland formula in the institution where it originated.[5] Sepsis also presents in non-traditional ways in the burn-injured patient.[1] I have summarized, for the non-burn physician and surgeon some of the key aspects of a recent consensus statement produced by the *American Burn Association *about organ-specific septic complications in the setting of burn injury.

A number of outcome indicators related to burn unit practice are coming into clearer focus. Several papers summarize this recent thinking and are reviewed here. First, renal failure has an incremental impact of mortality in any critical care unit population.[6] We now have data to suggest that similar concerns are true in burns. Burn units are often the site for management of soft tissue problems not specifically associated with extremes of temperature. The two most common problems of this type seen in burn practice are Toxic Epidermal Necrolysis (TEN) and soft tissue infections.[7,8] Both of these problems require the wound care expertise of burn unit personnel. Because these problems are relatively infrequent, outcome data related to Toxic Epidermal Necrolysis and soft tissue infection are hard to find or available only in early reports.[7,9] Recent papers attempt to bring outcomes of these important but infrequent problems into focus. The impact of burn verification on comparative outcomes in a geographic region is also reviewed.[10]

Finally, Western nations face an obesity epidemic.[11] The impact of obesity on functional outcome in burns has been compared to other complications. Recent reports shed light on this issue and are reviewed below.

## Materials and methods

This is a selected review of recent literature and summary statements of the *American Burn Association *provided for the physician or surgeon with an interest in injury who is not a regular burn unit practitioner or burn specialist. These papers are selected to provide an update summarizing key points in these recent reports.

### Resuscitation

**Figure 1:**
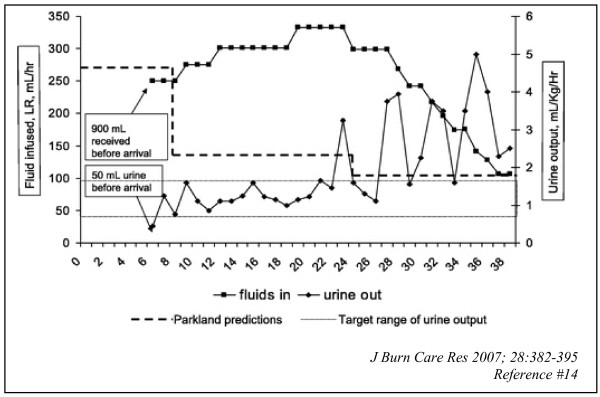
**Time course of fluid resuscitation for a 6 year-old boy (20 kg) with 33% TBSA scald burns**. He arrived at the burn center 6 hours post-injury, having received 900 ml of lactated Ringer's solution prior to arrival. Fluid resuscitation was started according to the Parkland forumula (heavy dashed line); nurses were instructed to maintain urine output between 0.9 and 1.8 ml/kg/h (dotted line). Initial resuscitation was close to Parkland guidelines, but beginning at about 10 hours post-burn, fluid requirements increased progressively until about 22 hours post-burn, when urine output finally began to rise, and fluids were tapered in a stepwise manner according to protocol. The patient reached his calculated maintenance fluid rate of 106 ml/h at hour 36. Total resuscitation received was 11.38 ml/kg/% TBSA. He had no difficulties with compartment syndromes or respiratory distress.

**Figure 2:**
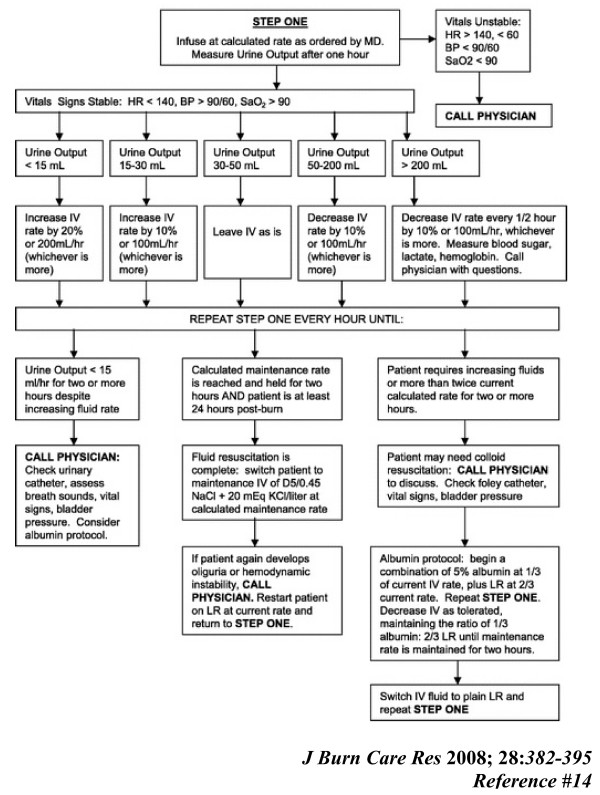
**Protocol for Fluid Resuscitation of the Adult Burn Patient (Begin LR Using Burn Center Fluid Resuscitation Calculations)**. Protocol for fluid resuscitation of adult burn patients. In response to requests from nursing, this protocol was developed to permit nursing staff to manage fluid resuscitation of acute burn patients. Initial fluid rates are calculated by the Parkland formula. Nurses begin hourly infusion, measure urine output, and adjust fluids according to patient response. Development of unstable vital signs, inadequate response to fluids, or persistently high fluid requirements prompt a call to the physician. A pathway to begin colloid replacement exists for patients who display increasing fluid requirements or develop evidence of torso compartment syndrome.

Fluid administration in the setting of burn injury, monitoring of efficacy and consensus recommendations are included in recent work published in the *Journal of Burn Care & Research*.[12] Blumetti and coworkers [13] from the University of Texas Southwestern in Dallas provide a 35 year retrospective and commentary on the present state of the Parkland formula. This standard for burn resuscitation has recently been critiqued in multiple studies and a recent editorial review by Saffle pointing out that patients frequently receive greater amounts of fluid than predicted.[14] He presents an example of resuscitation excess from his experience and presents a resuscitation program incorporating feedback, communication requirements and clinical targets (Figures 1 and 2). Accuracy and practicality of the Parkland formula are open to question.

Blumetti, et al. conducted a retrospective analysis of burn patients treated at Parkland Memorial Hospital Burn Center during a 15 year period from 1991 to 2005.[13] Included were burns in adults > 19% Total Body Surface Area (TBSA). In this adult group, adequate fluid resuscitation was defined as a urine output of 0.5 to 1.0 mL/kg/hr. Over resuscitation was defined as a urine output > 1.0 mL/kg/hr. In a review of nearly 500 patients, 43% received adequate resuscitation based on urine output criteria. Forty-eight percent were over resuscitated. There was no difference in complication rates or mortality regardless of over resuscitation versus adequate resuscitation. Patients were evaluated for inhalation injury with bronchoscopy. Contrary to reports from other centers, the amount of fluid required for adequate resuscitation based on target urine output was not different in patients with inhalation injury as opposed to those without this insult. While Ivy and others [15] demonstrated that intraabdominal hypertension and abdominal compartment syndrome commonly occurred in burn patients with volume resuscitation in excess of 250 mL/kg, the Parkland data reported here notes a 1% incidence of abdominal compartment syndrome even in burns exceeding 40% total body surface area where resuscitation volumes exceeded 250 mL/kg. In summary, even in the home of the Parkland formula, actual burn resuscitation frequently does not meet the standard set forth by this clinical strategy. Patients commonly received higher fluid volumes than predicted by the Parkland formula. The Parkland team recommended emphasis on calculated formula volumes only as a guide to initial resuscitation and the use of careful titration to urine output as the most important intervention.

Surrogate parameters for adequacy of resuscitation were discussed in two reports. Jeng and coworkers [16] focused on wound perfusion as a key factor promoting progression of burn depth and questioned whether parameters such as tissue and gastric PCO_2 _could provide more immediate data on efficiency of resuscitation than measurement of urine output and mean arterial pressure. Four patients with severe life-threatening burns (median 58% TBSA) and shock were enrolled in this study. All patients were adults with percent TBSA burns > 40%. Time between burn injury and arrival at the burn center was < 2 hours and patients were admitted directly to the burn center with admission mean arterial pressure < 70 mmHg. Patients with concomitant electrical injury, trauma or lack of consent within 24 hours were excluded. Patients were resuscitated to maintain oxygenation (> 90% saturation), urine output (30–50 mL/hr) and mean arterial pressure (> 70 mmHg). In these patients with large burns, crystalloid volumes used in the first 24 hours were very high, averaging 16.8 mL/kg/%TBSA burn, vastly exceeding the Parkland formula predictions. Even with this massive fluid administration, cyclic changes were noted in burn wound pH, PCO_2_, PaO_2_, gastric PCO_2_, gastric PO_2_, arterial pH and base deficit. When resuscitation parameters described above were compared to laser Doppler imaging, a standard used in this study to evaluate burn perfusion, changes in gastric PCO_2_, burn wound pH and burn wound PCO_2 _mimicked the changes in laser Doppler measured burn perfusion. Tissue resuscitation parameters showed statistically significant changes in perfusion 4 hours after the start of resuscitation while urine output did not change until 2 hours later. Remarkably, when burn wound perfusion was improved by interventions based on tissue parameters, the change did not translate into a measureable variation in hourly urine output. Use of tissue tonometry at both gastric and burn wound sites provided more rapid recognition of changes in resuscitation efficacy. It is important to note that the impact of these interventions on outcome cannot be demonstrated in this limited data set.

Batchinsky coworkers [17] at the US Army Institute of Surgical Research with collaboration from the University of Turku in Finland investigated heart rate variability and its relationship to cardiovascular regulation after burn injury. Investigators have noted in other settings of cardiovascular stress that loss of R to R interval complexity is seen. These investigators demonstrated abnormally low R to R interval complexity during early post-burn resuscitation in a series of 13 patients with mean total body surface area burn of 36%. All of these patients survived resuscitation. Progress through resuscitation was associated with improvement in R to R interval complexity and other evidence of improved end organ support. Nonlinear and frequency domain ECG analysis was employed. These results mimic those of other investigators studying trauma resuscitation, particularly the Vanderbilt group.[18]

A consensus statement has also been released from the *American Burn Association *regarding burn/shock resuscitation.[12] Notably, no "*standards*" for the approach to the resuscitation of burn injured patients exist from contemporary data. A number of "guidelines" are supported by evidence of lesser strength. "Guidelines" and "Options" from the *American Burn Association *are listed in Additional file 1 as published in the *Journal of Burn Care & Research*.[12]

Based on the strength of present evidence, there is no consensus regarding optimal fluid composition, rate of fluid administration and the role of colloid. No resuscitation parameters specific to individual patient fluid needs are better than routine hemodynamic endpoints and adequate urine output. Practitioners must be compulsive in providing adequate fluids but avoiding excessive resuscitation in any fluid program employed.

Three additional points of clarification regarding burn resuscitation should be made. First, many patients, particularly with burns < 20% TBSA may be candidates for oral resuscitation as an intact gastrointestinal tract is tolerant of large amounts of fluid administration. Enteral resuscitation should be considered particularly when resources are limited, an austere setting is encountered, and the patient is able to tolerate oral intake. Second, invasive hemodynamic monitors including central venous catheters and pulmonary artery catheters have been employed to optimize burn resuscitation in a variety of prospective and retrospective studies. Patients with invasive central hemodynamic monitors tended to have far more fluid administered without improvement in outcome. While invasive monitoring may be indicated for patients with special comorbidity or patients who fail to respond to resuscitation prescriptions, a blanket statement in favor of this approach cannot be made. Third, antioxidant therapies show promise in reduction of burn resuscitation fluid requirements and edema formation in a variety of preclinical trials. Unfortunately, patient data is limited and multicenter prospective validation has not been attempted.

### Sepsis and Infection in Burns

In a historic action, the *American Burn Association *convened a consensus conference on burn sepsis and infection using methodology recently employed by the *Society of Critical Care Medicine *and other critical care societies. [19-23] The conference and documents produced from it apply consensus-driven definitions of organ dysfunction and infection states as described in the general critical care population and modified these as appropriate to reflect the perturbation in burn injury. This work is important as the major cause of late death in the burn patient population is multiple organ dysfunction syndrome typically driven by infection. I will highlight findings of this consensus conference process below.

*The concept of Systemic Inflammatory Response Syndrome (SIRS) should not be applied to burn patients*.[20,24] While this concept is widely accepted and utilized in critical care practice and clinical trials, it has been widely criticized for being too inclusive and insufficiently specific to effectively identify a relevant inflammatory state. Burn patients frequently demonstrate characteristics of SIRS throughout the majority of hospitalization. Biochemical markers have also been evaluated but at present do not apply to the specific physiology of the burn patient.

*Sepsis is redefined in the burn patient population*.[19] Triggers in the burn injured patient are different than those in other critical care populations. As in general critical care practice, sepsis is a condition warranting empiric antibiotics and a search for infection during that short course of empiric therapy. Definitions for sepsis in the burn patient are given in Additional file 2.

*The concept of severe sepsis, the intervening state between sepsis and septic shock was dropped *as the conference attendees felt that as a distinct state between sepsis and septic shock, severe sepsis is not regularly seen. *Septic shock *definitions from recent consensus conferences including the **Surviving Sepsis Campaign **and consensus work of the major critical care societies are retained.[25]

*Septic shock *is defined as sepsis-induced hypotension persisting despite adequate fluid resuscitation. Sepsis-induced hypotension is defined as a systolic blood pressure (SBP) < 90 mmHg or mean arterial pressure < 70 mmHg or a SBP decrease > 40 mmHg or < 2 SD below normal for age in the absence of other causes of hypotension. Sepsis-induced tissue hypoperfusion is defined as septic shock, an elevated lactate or oliguria.[25]

*Smoke inhalation injury *by anatomic definition occurs below the glottis and is caused by products of combustion. Diagnosis requires history of exposure to products of combustion and bronchoscopy revealing carbonaceous material or signs of edema/ulceration. Smoke inhalation injury can occur with or without detection of products such as cyanide or carbon monoxide. Anatomic injury, however, is the hallmark of smoke inhalation injury. Bronchoscopy is the "*gold standard*" for diagnosis.

*Pneumonia*, a common complication of inhalation injury, is defined similar to previous consensus conferences by critical care and respiratory societies.[26,27] The ABA Consensus Group did make statements regarding positive microbiology. Greater than 10^5 ^organisms on a tracheal aspirate, bronchoalveolar lavage with ≥ 10^4 ^organisms and protected bronchial brushings with > 10^3 ^organisms are general criteria for positive microbiology in the setting of burn injury. The burn literature supports discontinuation of antibiotics where microbiologic thresholds are not met. The Clinical Pulmonary Infection Score was briefly discussed and felt to be insufficient to predict ventilator-associated pneumonia in the burn victim. Clinical suspicion of ventilator-associated pneumonia must be verified by quantitative culture results.

Definitions for bloodstream and catheter-related bloodstreams infections are accepted as defined previously.[28,29] Catheter colonization is seen where growth of organisms from a catheter segment is identified by semiquantitative or quantitative culture. A catheter-related bloodstream infection reflects identification of the identical organism in a blood culture and a semiquantitative or quantitative culture from a catheter segment. Clinical symptoms of bloodstream infection should also be present without any other apparent source of infection. Finally, exit site infection is defined as erythema, tenderness, induration or purulence within 2 cm of the exit site of a catheter. Blood cultures should ideally include quantitative technique with a specimen of ≥ 10 ml. Blood cultures must also reflect recognized pathogens not usually regarded as skin contaminants cultured from one or more blood cultures. An organism cultured from the blood to reflect a primary bloodstream infection is not related to infection at another site.

Definitions of wound-related complications were also assembled. [30-32]***Wound colonization ***is present with bacteria on the wound surface at low concentrations. ***Wound infection ***is present with high concentrations of bacteria in the wound and wound eschar (> 10^5 ^bacteria/gram tissue). ***Invasive infection ***occurs with high concentration of pathogens (> 10^5 ^bacteria/gram tissue) and changes such as separation of eschar or skin grafts, invasion of adjacent unburned tissue or development of sepsis as defined above. ***Cellulitis ***is seen with bacteria in the wound or wound eschar at high concentrations and advancing erythema, induration, warmth and tenderness of surrounding tissues. Sepsis, as defined above, must be present. ***Necrotizing fasciitis ***is an aggressive, invasive infection with necrosis of tissues beneath the skin. Among objective diagnostic criteria are biopsy and swab culture techniques. None of these are ideal. Clinical correlates include systemic changes, premature separation of eschar and unexplained tissue loss or changes in the depth of wounds. *Pseudomonas aeruginosa *is a frequent colonizing organism in burn and other soft tissue wounds. The yellow/green exudate of pseudomonas does not reflect invasive infection. When changes consistent with deep tissue injury and systemic changes including organ dysfunction are seen, aggressive antibiotic therapy and surgical debridement are warranted.[19]

This rich document summarizes much of the contemporary thinking on infectious complications in the setting of burns and soft tissue injury. The authors hope that this standardization of reporting for infectious complications and associated organ dysfunction with burn injury leads to improved study design and evolution of new therapies for these patients.

### Obesity

Two papers examine the impact of obesity on disability after burn injury and mortality related to this form of trauma. In the first paper, Carpenter and coworkers [33] review the *National Burn Registry *from 2000 to 2006. This review included over 100,000 patients. These investigators stratified patients by their identification as obese or non-obese in the Registry. Two outcome measures examined were length of stay (< 7 days or ≥ 7 days) and mortality. Obese patients were far more likely to have to have a length of stay > 7 days and to die following burn admission. Of factors gathered in the Registry, obesity was more than 4 times as likely to be associated with length of stay 7 days or greater (*p *< 0.0001). In terms of magnitude of impact on length of stay, obesity is similar to coronary artery disease, hypertension and alcohol abuse as a risk factor for length of stay 7 days or more. Obesity increased mortality 2.6-fold in this large data sample (*p *= 0.001). The impact of obesity on mortality was similar to that of coronary artery disease and hypertension. With respect to length of stay and mortality, pneumonia was a more powerful predictor than obesity and the other factors discussed.

Obesity is common in the United States. Literature cited by these authors notes that 32% of the American population may now be considered to be obese compared with 20% one decade ago. [34-37] Remarkably, in this data set, the total number of patients indicated as obese is only 672 out of over 100,000 entries. Thus, a significant under representation and biased representation of obesity is possible in the *National Burn Registry*. The burn size and characteristics in obese as opposed to non-obese patients are not discussed. While I do not argue with the impact of obesity on outcomes in burn injury as presented here, it is clear that a stronger emphasis on population of data fields related to nutrition status will enhance the power of the *National Burn Registry *to effectively evaluate the impact of obesity on burn outcomes.

Farrell and coworkers [38] from the Loyola Burn Center examined the impact of obesity on functional outcomes in a more tightly characterized acute burn population. Recognizing that factors other than body mass index may affect discharge disposition in patients with burn injury, these investigators evaluated the impact of age, TBSA burn, inhalation injury and Body Mass Index (BMI) on discharge disposition. This study was much smaller than that previously reported with only 221 patients age 16 or older. However, the consistency of recording is greater than the massive database of the *National Burn Registry*. The Loyola investigators recorded not only metabolic data but also Functional Independence Measure (FIM) scores for patients evaluated.

Using a Classification and Regression Tree (CART), nonparametric analysis methodology, these investigators found that the strongest predictor of discharge disposition was TBSA burn. Over 80% of patients with < 30.75% TBSA burns returned directly home after hospitalization. Only 27.6% of patients with > 30.75% TBSA burns were discharged to home. In patients with < 30.75% TBSA burns, age also predicted discharge disposition. Patients younger than 57.5 years were more likely to go home (90%) compared with patients older than 57.5 years (47.5%). In patients older than 57.5 years with < 30.75% TBSA burns, BMI has a role in predicting discharge disposition. In this population, BMI > 27.4 suggested decreased likelihood to return home (21.4%) while patients with a lower BMI (< 27.4) were more likely to return home (65%). Age, inhalation injury and BMI did not have a role in predicting discharge in patients with larger burns.

Particular attention was paid to Functional Independence Measure (FIM) transfer scores. The strongest predictor of FIM transfer scores was the size of the burn. Patients with < 22.5% TBSA burns had a higher FIM score than patients with > 22.5% TBSA burns. In older patients with smaller burns, BMI was a factor. Patients with a BMI < 31.25 had a higher average FIM score than larger patients.

The CART regression model used by the Loyola team is a tool permitting clinicians to determine variables contributing to patient outcome in the face of specific patient characteristics. Not surprisingly, size of injury was the strongest predictor of outcome. [39-41] However, in larger burns, female gender also predicts poorer outcome than males. Older patients with smaller burns most acutely feel the impact of obesity where reduced function is noted in the larger patient. Finally, this work confirms the adverse effect of increasing age on functional outcomes after burns.

While 221 subjects is significant given the number of data points collected, many of the individual comparisons in this multiple regression technique contain small numbers of patients. Insurance and other social status concerns could also affect disposition at discharge. These potential sources of bias cannot be eliminated in this retrospective work.

### Pulmonary Injury

Respiratory failure in burns is generally characterized by hypoxemia with evolution to Acute Lung Injury or Acute Respiratory Distress Syndrome (ARDS).[42] Even in patients without defined inhalation injury, the presence of ARDS is associated with poorer outcome. Where smoke inhalation and ARDS are combined, in the pediatric population a mortality in excess of 50% is noted.[43] While there is no consensus regarding the optimal ventilatory strategy, two recent papers discuss potential adjuvant therapies. A report from the Cincinnati Shriners Hospital for Children evaluates an anti-inflammatory pulmonary enteral nutrition formula which has been previously used in adults.[43] This is a retrospective review examining patients receiving a specialized enteral formula in the setting of respiratory failure after burn injury. Mean age of this patient group was approximately 5 years. Patients included had a PaO_2_/FiO_2 _ratio of < 200 mmHg at the time of specialized nutrition support. Median burn size was 36% TBSA with 24% TBSA full thickness. Seventeen of 19 patients ultimately survived. In some cases, multiple tube thoracostomies and advanced ventilator support were required. In addition to improvements seen in respiratory function parameters, chemistries including BUN, creatinine, sodium and potassium also improved with administration of the specialized nutrition support program. This limited retrospective data set is presented to support further evaluation of specialized nutrition support for burn injured patients.

There is a clear physiologic rationale for the specific nutrition intervention provided.[43,44] The formula tested provides a high quantity of protein and moderately low carbohydrates.[45] Eicosapentaenoic acid, gamma-linolenic acid and antioxidants are added for anti-inflammatory properties and to oppose the tendency toward oxidative stress in the burn injured patient with lung injury. [46-49] Elsewhere in the critical care literature, however, there has been a movement away from immunonutrition due to inability to predict, in a consistent way, the appropriate timing for proinflammatory or anti-inflammatory interventions.[50] Clearly, in this retrospective database, many interventions, particularly ventilator strategies which could affect the outcome of lung injury in burn injured patients, are not discussed. Perhaps most important, we lack a consistent understanding of the physiology of inflammation in lung injury and how to assess the inflammatory state of a patient when this anti-inflammatory material is given. Caution is advised.

Holt and coworkers [51] from the University of Utah examined another adjuvant therapy for inhalation injury, a combination protocol of inhaled heparin/N-acetylcystine. Again, a retrospective database was employed. Sixty-two of 150 patients admitted with inhalation injury from 1999 to 2005 were reviewed. Inhalation injury was based on confirmatory bronchoscopy, clinical suspicion or elevated levels of carboxyhemoglobin. The authors do not indicate the proportional use of each diagnostic criteria. Demographic and pulmonary data were reviewed.

The heparin/N-acetylcystine protocol utilized at this burn center was initiated at the time of admission but at the attending physician's discretion. Patients placed on this protocol received inhaled heparin (5000 U/l mL), N-acetylcystine (3 mL of 20% solution) and albuterol (2.5 mg of 0.083% solution per 3 mL) every 4 hours for the first 7 days after admission or until extubation. No difference in key physiologic or clinical outcomes was noted. The authors do report better PaO_2 _on day #1 and day #3 of the heparin/N-acetylcystine protocol compared to patients who did not receive these medications. By 72 hours, this difference had disappeared. Overall, mortality, incidence of pneumonia and resource consumption was not affected.

Management of inhalation injury consists of supportive care. This may include mechanical ventilation with supplemental oxygen therapy and pulmonary toilet either through catheter means or bronchoscopic interventions. Herndon and the Galveston group have investigated in preclinical and clinical material the impact of strategies to reduce inflammation and free radical formation with combinations of heparin/N-acetylcystine. [52-55] The Utah group was unable to replicate the success of the Galveston program with inhaled heparin/N-acetylcystine for reasons which remain unclear. It should be noted that the data reported by the Galveston group favoring the use of heparin/N-acetylcystine came from a pediatric patient population where the Utah group studied both adults and children. In addition, the Utah data is retrospective and in the discussion, writers admit that not all chart data was complete. At best, we are left with a call for additional prospective, multicenter data evaluating anti-inflammatory strategies in the setting of lung injury and smoke inhalation.

### Outcomes

For over half a century, investigators have sought predictive indices for outcomes in burn injury. Perhaps the best known is the Baux rule, a simple sum of patient age and total body surface area suffering 2^nd ^and 3^rd ^degree burns.[56] This index has been and continues to receive attention. In fact, the Baux rule was recently addressed using patient registry data from the *American Burn Association*.[57]

Two more recent articles detail burn outcome. The more recent comes from the National Burn Repository which published a 10 year review in 2006.[58] In all, over 125,000 acute burn admissions to United States' burn centers were described. Seventy percent of hospital admissions were male (mean age 33 years). Infants accounted for 10% of cases and patients aged ≥ 70 years comprised 8.5% of cases. Thirty-two percent of admissions were < 20 years old. Sixty percent of patients were 5 to 50 years olds. Sixty-two percent of patients had a total burn size of < 10% of total body surface area with 21% having burn size between 10–19.9% of the total body surface area. Only 10% of patients had burn size > 30% total body surface area. Inhalation injury was reported in 6.5% of patients. In patients sustaining inhalation injury, mortality was 30% as opposed to 5% for the patient group as a whole. Thus, inhalation injury has a disproportionate effect on mortality following burns.

Flame and scald burns accounted for 78% of total cases with the largest fraction of injuries occurring in the home (43%).[58] Work-related injuries comprised 17% of all cases. Survival in the overall study cohort has remained at approximately 95%. Deaths from burn injury increased with advanced age and burn size and in the presence of inhalation injury. The leading cause of death was multiple organ failure. Most frequent complications were pneumonia, wound infection and cellulitis. During the 10 year period from 1995 to 2005, the average length of stay declined from 13 to 8 days.

A second review of over 1600 patients admitted to the Massachusetts General Hospital and the Schriners' Burn Institute in Boston was published in early 1998.[59] Logistic regression analysis was employed to develop probability estimates for mortality based on a small set of well-defined variables. Mean burn size and survival were similar to the larger report above. Three risk factors for death were identified: age > 60 years; total body surface area burn > 40% and inhalation injury. The mortality formula developed from these reports predicts 0.3%, 3%, 33% or 90% mortality depending on whether 0, 1, 2 or 3 risk factors are present respectively.

A more recent report from a Canadian regional burn center was recently published in the *Journal of Trauma*.[60] An accompanying report also appears in the *Journal of Burn Care and Research*.[61] The Canadian investigators proposed predictors including characteristics of the burn and APACHE II Score as predictive prediction of outcome. The score coined by these investigators (FLAMES) was derived in a population seen between 1991 and 1995 and validated in a larger population treated between 1995 and 2003. The FLAMES Score including fatality by longevity, APACHE II Score, gender and extent of burn had an excellent receiver operating characteristic curve of 0.97. Comparable results were obtained in the development and validation populations. Two interesting observations can be made from this data. First, inhalation injury had no impact on outcome. In fact, the number of patients with documented inhalation injury is exceedingly small. In this respect, this dataset is unlike that seen in American burn centers[60]. A second observation stems from a report published by the same investigators about improvement in survival over time in patients treated at the same burn center.[61] Thus, consistency in clinical practice may not have been present during the interval of this work. Finally, I note that the APACHE II Score, as employed by these investigators, was developed in a population which did not include burn patients.

Mustonen and Vuola [62] from Helsinki review burn ICU outcomes with acute renal failure in a population of over 1300 patients admitted between November 1988 and December 2001. These authors used a liberal definition of "*acute renal failure*", defining it as a rise in serum creatinine of > 120 μmol/l (1.4 mg/dl) or a two-fold rise in creatinine by more than 100 μmol/l (1.1 mg/dl) during one day. These authors were very aggressive with initiation of renal replacement therapy. They began renal replacement therapy with serum creatinine > 180 μmol/l (2 mg/dl), urea > 30 μmol/l, anasarca or pulmonary edema without response to diuretics. Of 238 patients admitted to the intensive care unit, 39% suffered some form of renal injury as defined above and 13.4% of patients required renal replacement therapy. Examined in the burn population as a whole, 2.3% of patients required renal replacement therapy.

After stratifying patients based on presence or absence of acute renal failure, the authors note that mortality of patients without renal failure was 6.9% but that of patients with renal failure was 44.1%. In the more severely affected population, those patients receiving renal replacement therapy, mortality was 62.5%. Not surprisingly, burn size was significantly larger in patients with acute renal failure (40.2%) than in patients without acute renal failure (25.7%). Consistent with the critical care literature in general, if the patient survives in ICU, kidney recovery occurs. Only one patient in this series had to continue intermittent hemodialysis after discharge from the hospital. None of the patients who survived after acute renal failure with renal replacement therapy required dialysis at one year.

The Helsinki data is consistent with the broader critical care literature in that mortality associated with acute renal insufficiency, particularly if dialysis is required, is 50–60%. [63-65] If patients survive hospitalization, renal insufficiency tends to resolve, though biochemical abnormality may remain. The low overall incidence of renal insufficiency in this population reflects the effectiveness of the Parkland formula-based resuscitation utilized by these practitioners. Though infrequent, rhabodomyolysis predicted poor outcome in these patients.[66]

Toxic Epidermal Necrolysis (TEN), originally described in the 1950s is now a growing part of burn center practice.[67] While pathogenesis remains unclear, TEN is thought to be one of a continuum of disorders associated with epidermal detachment associated with inflammation and denudation of mucosal surfaces.[68,69] The most common trigger for TEN is thought to be drug exposure with anticonvulsants, sulfa drugs and allopurinol the frequently implicated agents. Other disorders in the series of cutaneous drug reaction syndromes include Stevens Johnson Syndrome which is the mildest manifestation of this group of disorders and the intermediate severity state of Stevens Johnson Syndrome-Toxic Epidermal Necrolysis (SJS-TEN). These forms of illness are distinguished by extent of epidermal detachment involving > 30%, 10–30% or < 10% of the TBSA in TEN, SJS-TEN and SJS. Outcome appears to vary according to diagnosis (and TBSA involvement). Mortality in TEN is approximately 30% while that of SJS is < 5%. The primary cause of death is infection and multiorgan failure. The likelihood of developing TEN is significantly higher in patients with HIV related disorders than the normal population.[68,69]

General treatment begins with withdrawal of any offending drug.[68] Medication intake for up to a month prior to the onset of symptoms must be examined for potential offending agents. Skin care involves removal of nonviable epidermis and some form of coverage. Some authors recommend the use of biologic dressings including cryoperserved cutaneous allografts, porcine zenografts, collagen-based substitutes or amnion-based prostheses. Others use the involved native epidermis as a biologic dressing with replacement dressings only on exposed dermis. The literature holds no consensus regarding the value of early removal of involved epidermis. Ocular involvement requires topical lubrication, steroid drops and release of symblepharon.

**Figure 3:**
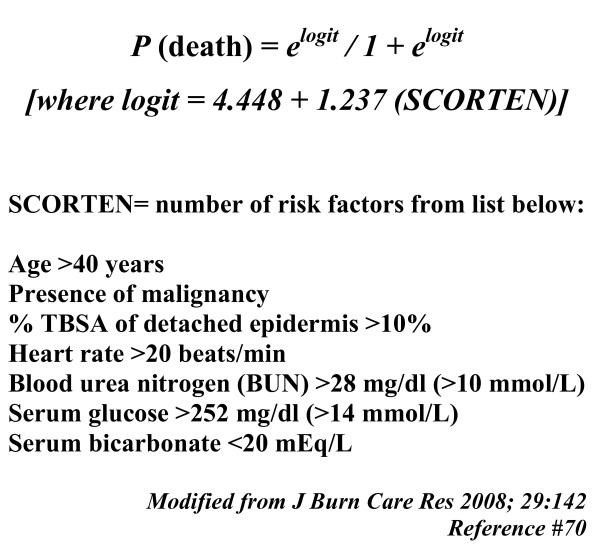
**SCORTEN Mortality and Risk Variables**.

Outcome is predicted by the SCORTEN system which uses variables present during the first 24 hours after admission to estimate the severity of TEN and predict mortality.[70,71] Originally described in a series of patients with cutaneous disorders ranging from SJS to TEN, SCORTEN gives each risk factor a score of 1 and utilizes the sum of these values so that a higher score is associated with greater mortality. SCORTEN is based on a logistic regression study deriving a predictive death equation. The figure and table below indicate the SCORTEN risk variables and outcomes. Each variable is one SCORTEN point. (Figure 3 and Additional file 3)

The Ross Tilley Burn Centre recently evaluated the SCORTEN system in a consecutive series of patients admitted between April 5, 1995 and June 10, 2006.[70] Good correlation between actual outcomes and predicted mortality from the SCORTEN relationship was identified. This is remarkable given the significant amount of time over which these cases were collected. Another recent study involved 144 patients treated between 1993 and 2003 where SCORTEN was calculated daily during the first 5 days of admission.[72] The discrimative power of SCORTEN improved with repeated calculation. Thus, SCORTEN may be even more accurate if calculated in a serial fashion rather than basing it on variables present on the first day of hospitalization, as originally described.

A number of treatment strategies for TEN have been proposed.[68,73] However, given the infrequent presentation of these patients, randomized, prospective data is rare and multicenter trials may ultimately be required to provide consensus-driven treatment strategies. The lack of definitive understanding of the pathology of TEN also hampers therapies based on an understanding of mechanisms of disease.

Another dimension of practice in many burn centers is management of complex wounds including soft tissue infections. Saffle and coworkers [74] from the University of Utah investigate the outcome associated with Fournier's gangrene, a necrotizing infection associated with genitalia, perineum or perianal tissues. While burn centers frequently care for these patients, Saffle and coworkers compared burn center results to those obtained in other settings. [74-77]

A review of charts took place at the Utah Burn Center from 1992 to 1995 and identified 30 patients with complex perineal infections. Among demographic data, male gender (67%), the presence of diabetes and referral from outside hospitals were common. Many of these patients were morbidly obese and used systemic immunosuppressing agents including corticosteroids. Liver disease and renal insufficiency were also common. Standard of care was initial operative debridement within 24 hours of admission. Patients were subsequently returned to the operating room every 2 to 3 days until debridement was complete and remaining tissue appeared viable. Burn dressings with topical antibiotics were employed to compliment systemic antimicrobial therapy. Skin grafting or local tissue flaps were commonly used for wound closure. Patients required a mean of 4.1 surgical procedures. Definitive wound closure was obtained in 72% of patients prior to discharge. Patients with open wounds were treated with negative pressure dressings or wound packing until secondary healing occurred. Mean length of hospitalization was 25 days. Consistent with other reports, five patients died in this series for a mortality of 16.6%.[78] Hyperbaric oxygen was not available in this center and not employed in management of this series of patients.[79]

There is a wide range of mortality reported for complex perineal infections in the literature. Due to the uncommon nature of this presentation, large data sets do not exist. The role of hyperbaric oxygen remains unclear but this dataset suggests that aggressive surgery, broad organ system report and antibiotic therapy provide equivalent outcomes where hyperbaric oxygen is not available.[79] Further, these authors note that diverting procedures including suprapubic cystostomy, colostomy and orchiectomy are generally not required and that burn centers can provide adequate reconstruction for these complex problems.

Development of burn centers in the United States has been associated with a verification process to standardize and optimize overall quality of care delivered.[80,81] Burn centers have been demonstrated to improve survival and decrease resource consumption. Palmieri and coworkers [80] examined the difference in outcomes between burn centers receiving verification from the *American Burn Association *and the *American College of Surgeons *as opposed to burn centers which were not verified. Using the discharge database from the state of California, 5 verified burn centers were compared with 12 non-verified burn centers. In 2003, 2867 patients were admitted to burn centers; over 1600 of these patients were admitted to non-verified centers. Non-verified centers admitted 132 patients per center while verified centers admitted 244 patients per center. Verified centers admitted more patients with large burns, burns requiring complex reconstruction and more patients requiring critical care support including mechanical ventilation. Verified burn centers also performed fewer operations than non-verified centers. More patients from verified centers were able to return home while additional rehabilitation care was required in patients from non-verified centers. Mortality, however, was 3% in non-verified burn centers and 4% in verified burn centers.

While the lack of difference between morality in verified and non-verified burn centers is encouraging, significant differences in resource consumption including operative procedures, post-discharge destination and median hospital charge support the use of verified burn centers in the management of burn-injured patients. Clearly, the population seen in verified and non-verified burn centers is different and this report details resource consumption issues which may be better addressed in a regionalized burn care system.[82,83]

## Conclusion

• The burn patient is easily over resuscitated. Practitioners must be willing to reduce fluid prescriptions when signs of adequate perfusion are present. Currently, adequate vital signs and urine output are the *"gold standard" *for perfusion assessment.

• The burn-injured patient does not fit traditional definitions of Systemic Inflammatory Response Syndrome (SIRS) and sepsis must be redefined based on the physiologic characteristics of burn injury. In addition, the burn patient is at risk for soft tissue infections and burn wound infection which have been better defined.

• Obesity is a marker for increased resource consumption in the setting of burn injury. Impact of obesity on mortality is similar to that of coronary artery disease and hypertension.

• *"Gold standard" *therapy for inhalation injury remains lung protective mechanical ventilation. Specialized nutrition and medical therapies have been evaluated but widespread consensus regarding their value is absent.

• A number of factors are predictive of mortality in burn injury. Burn size, presence or absence of inhalation injury and extremes of age have been previously reported.

• Renal failure and insufficiency is a marker for poor outcome following burn injury.

• Support for use of burn centers in management of soft tissue problems outside traditional burn injuries such as Toxic Epidermal Necrolysis and soft tissue infections can be obtained from recent papers.

• Use of verified burn centers reduces cost of therapy for burn injury.

## Competing interests

The author declares that they have no competing interests.

## Supplementary Material

Additional file 1 **Table S1. **Burn Shock Resuscitation. Click here for file

Additional file 2 **Table S2. **Description: Definitions for Sepsis in the Burn Patient. Click here for file

Additional file 3 **Table S3. **Predicting Mortality in TENS Based on SCORTEN. Click here for file
