# Supplementary material for: Management of burn injuries – recent developments in resuscitation, infection control and outcomes research
Source: Scand J Trauma Resusc Emerg Med. 2009 Mar 11;17:14. doi: 10.1186/1757-7241-17-14 (PMC2666628; doi:10.1186/1757-7241-17-14)
Supplement: Additional file 1 — Table S1. Burn Shock Resuscitation. [file 1757-7241-17-14-S1.doc]

**Table 1**

**Burn Shock Resuscitation**

**Guidelines:**

- Adults and children with burns greater than 20% Total Body Surface Area (TBSA) should undergo formal fluid resuscitation using estimates based on body size and surface area burned.

- Common formulas used to initiate resuscitation estimate a crystalloid need of 2 to 4 mL/kg body weight/%

TBSA during the first 24 hours.

-Fluid resuscitation, regardless of solution type or estimated need, should be titrated to maintain a urine output

of approximately 0.5-1.0 mL/kg/hr in adults and 1.0-1.5 mL/kg/hr in children.

- Maintenance fluids should be administered to children in addition to their calculated fluid requirements

caused by injury.

- Increased volume requirements can be anticipated in patients with full-thickness injuries, inhalation injury and a delay in resuscitation.

**Options:**

- The addition of colloid-containing fluid following burn injury, especially after the first 12 to 24 hours

postburn, may decrease overall fluid requirements.

- Oral resuscitation should be considered in awake alert patients with moderately sized burns and is worthy of

further study.

- Hypertonic saline should be reserved to providers experienced in this approach. Plasma sodium

concentrations should be closely monitored to avoid excessive hypernatremia.

- Administration of high-dose ascorbic acid may decrease overall fluid requirements, and is worthy of further study.

*J Burn Care Res 2008; 29:257-266*

*Reference #12*
