# Supplementary material for: Management of burn injuries – recent developments in resuscitation, infection control and outcomes research
Source: Scand J Trauma Resusc Emerg Med. 2009 Mar 11;17:14. doi: 10.1186/1757-7241-17-14 (PMC2666628; doi:10.1186/1757-7241-17-14)
Supplement: Additional file 2 — Table S2. Description: Definitions for Sepsis in the Burn Patient. [file 1757-7241-17-14-S2.doc]

**Table 2**

**Definitions for Sepsis in the Burn Patient**

-Temperature >39° or <36.5° (centigrade)

-Progressive tachycardia –

Adults >110 breaths/min

Children >2 SD above age-specific norms *(85% age adjusted max heart rate)*

-Progressive tachypnea

Adults >25 breaths/min not ventilated

Minute ventilation >12 l/min ventilated

Children >2 SD above age-specific norms *(85% age-adjusted max respiratory rate)*

-Thrombocytopenia *(will not apply until 3 days after initial resuscitation)*

Adults <100,000/microliter

Children <2 SD below age specific norms

-Hyperglycemia *(in absence of preexisting diabetes mellitus)*

Untreated plasma glucose >200 mg/dl (or equivalent mM/L)

Insulin resistance-examples include:

>7 units of insulin/hr intravenous drip [adults]

Significant resistance to insulin *(>25% increase in insulin requirements over 24 hours)*

-Inability to continue enteral feedings >24 hours

Abdominal distension

Enteral feeding intolerance *(residual >150 ml/hr in children or two times feeding rate in adults)*

Uncontrollable diarrhea *(>2500 ml/d for adults or >400 ml/d in children)*

***In addition, it is required that a documented infection is identified:***

-Culture positive infection, or

-Pathologic tissue source identified, or

-Clinical response to antimicrobials

***J Burn Care Res 2007; 28:776-790***

***Reference #19***
