# Supplementary material for: Management of burn injuries – recent developments in resuscitation, infection control and outcomes research
Source: Scand J Trauma Resusc Emerg Med. 2009 Mar 11;17:14. doi: 10.1186/1757-7241-17-14 (PMC2666628; doi:10.1186/1757-7241-17-14)
Supplement: Additional file 3 — Table S3. Predicting Mortality in TENS Based on SCORTEN. [file 1757-7241-17-14-S3.doc]

**Table 3**

| **Predicting Mortality in TENS Based on SCORTEN** | |
| --- | --- |
| **SCORTEN Value** | **Predicted Mortality Rate** |
| **0-1** | **3.2%** |
| **2** | **12.1%** |
| **3** | **32.4%** |
| **4** | **62.2%** |
| **5** | **85.5%** |
| **>6** | **95.0%** |
| ***J Burn Care Res 2008; 29:272***  ***Reference #68*** | |
